# Geriatric critical care assessment: key considerations

**DOI:** 10.1007/s41999-026-01478-y

**Published:** 2026-04-30

**Authors:** James D. van Oppen, Ben Bloom, Simon Conroy

**Affiliations:** 1 Centre for Urgent and Emergency Care Research, University of Sheffield, Sheffield, S1 4DA UK; 2 Emergency Medicine, Barts Health NHS Trust, Whitechapel Road, London, UK; 3 Blizard Institute for Neuroscience, Surgery, & Trauma, Barts & The London School of Medicine, Turner Street, London, UK; 4 Geriatric Medicine, Barts Health NHS Trust, Whitechapel Road, London, UK; 5 Wolfson Institute of Population Health, Queen Mary University of London, London, UK

**Keywords:** Frailty, Critical illness, Comprehensive geriatric assessment, Person-centred care

## Abstract

Frailty, multimorbidity, and altered physiology each and together shape the presentation, management, and outcomes for older people with critical illness. This narrative review of the field argues that the structures and processes of geriatric critical care must align with the outcomes that matter to older people living with frailty. This extends the scope of consideration beyond assumptions which may necessarily be restorative or curative, to focus on those interventions which are most achievable and desirable. Frailty is a stronger predictor of mortality, functional decline, and health-related quality of life than age alone. Yet frailty screening changes nothing, unless there is a corresponding change in clinical approach. We review the ABCDE-Frailty framework, operationalising frailty-attuned assessment in standard emergency evaluation using the 5Ms: Mind, Mobility, Medications, Multicomplexity, and Matters most. Person-centred decision-making, incorporating shared value- and goal-based understanding, provides the ethical and clinical foundation for proportionate treatment in critical situations. Avoiding non-beneficial intervention is not therapeutic nihilism but rather its opposite: care directed towards dignity, function, and the outcomes people themselves would choose. A paradigm shift is required, from survival-centred to person-centred, frailty-attuned geriatric critical care.

## Key summary points

**Aim:**

This is a narrative review of the literature concerning the emergency clinical assessment for older people with critical illness or injury.

**Findings:**

Frailty is a stronger independent predictor of mortality and functional outcomes from critical care than age alone. Clinical structures and processes can be frailty-attuned to account for these outcomes by considering value-based domains alongside conventional assessment.

**Message:**

Optimising critical care for older people living with frailty requires a paradigm shift from survival-centred to person-centred, frailty-attuned assessment and decision-making, in which recognising what matters most to the individual is both clinically and ethically intrinsic.

## Introduction: starting with the end in mind

Patient values and realistic outcomes fundamentally influence care delivery. Donabedian (1919–2000, a physician and founder of the study of quality in health care and medical outcomes research) described how structures (concrete resources, such as clinical staff or facilities) and processes (interactions between clinicians and patients) lead to outcomes. In many Western healthcare settings, the assumption in the context of a critical illness is that restorative or curative outcomes are the priority. This is entirely appropriate (for the most part) for people who are not frail. For instance, if you present with meningococcal septicaemia, you want rapid curative treatment to return as swiftly as possible to your usual activities. However, as people develop more severe frailty, their worldview shifts through a combination of loss/bereavement and a shrinking physical environment. As a consequence, their outcomes and priorities for life and healthcare change. The likelihood of medicine being able to achieve survival and recovery to baseline is at the same time markedly reduced when frailty is more severe. Instead of longevity at any price, preservation of function, dignity, comfort, and time at home with family becomes more important.

Given this change in outcomes and outcome goals, Donabedian teaches us that the structure and process of healthcare should alter accordingly. This paper responds to the question of whether in geriatric assessment of critically ill patients there is time for more than frailty screening. The answer is that yes, there is, and there needs to be. The structures and processes of healthcare need to adapt to align with the outcomes that matter to older people living with frailty. Assessing frailty is critical, as it alerts clinicians and healthcare systems to the fundamentally different characteristics of older people living with frailty, but subsequent to this there are ramifications for the entire approach. Indeed, simply screening for frailty changes nothing, but the response to that screening potentially changes everything.

## Outcomes and trajectories in geriatric critical care

Outcomes following critical illness in older adults depend on a complex interaction between acute physiological derangement, premorbid health status, and the intensity of organ support delivered, which itself is correlated to predicted outcome. In very old populations (≥ 80 years), age alone is a poor discriminator of outcome [1]. Frailty, illness severity, and treatment limitations are more informative [2].

### ICU populations

Large registry and cohort studies have shown that adults aged ≥ 80 years represent a substantial proportion of ICU admissions, with important international variation in access, case mix, and outcomes. In Australia and New Zealand, early registry data demonstrated a rapid rise in admissions of very old patients, who had higher comorbidity burdens, increased ICU and hospital mortality, and lower rates of discharge home, but with approximately 80% surviving to hospital discharge [3].

### Mortality

More recent analyses from Australia and New Zealand confirmed that this group now accounts for around 15% of ICU admissions, with persistently higher mortality and greater need for institutional care after discharge, despite substantial improvements in risk-adjusted survival over time [4]. Cross-national comparisons between the UK and USA further highlight how resource availability influences admission patterns and outcomes, with fewer very old patients admitted in the UK, higher illness severity at admission, and marked differences in discharge practices [5].

Prospective European studies have consistently demonstrated that frailty and premorbid functional status are central determinants of outcome in very old critically ill patients. The VIP-1 study showed that frailty (Clinical Frailty Scale ≥ 5) was present in over 40% of patients aged ≥ 80 years and was independently associated with increased ICU and 30-day mortality, regardless of illness severity or treatment intensity [6]. Patients with high frailty (Clinical Frailty Scale [CFS] ≥ 5) had significantly worse outcomes than those with low frailty, with hazard ratios for 30-day mortality of ~ 1.5 even after adjustment for illness severity. UK-specific data from the VIP cohorts back up this difference, with 30-day survival falling from 68% in fit patients to 58% in frail patients [7].

However, even within frail patients, outcomes are not uniformly poor. In UK data, approximately three out of five patients with mild–moderate frailty (CFS 5–6) survived to 30 days, indicating that frailty alone is an insufficient determinant of survival.

The VIP-2 study extended these findings by demonstrating that frailty, cognitive impairment, and disability were highly prevalent and closely correlated, with frailty remaining an independent predictor of 30-day survival after adjustment for SOFA score and admission diagnosis [1]. UK analyses of VIP cohorts confirmed a graded association between increasing frailty and reduced 30-day survival, reinforcing the prognostic value of CFS within the NHS context [7]. More recently, clustering analyses of VIP-2 data have identified distinct phenotypes based on combinations of frailty, multimorbidity, cognitive impairment, and organ dysfunction, with wide variation in short-term mortality between groups [8]. These studies, which enrolled nearly 9000 very old patients, demonstrated that short-term mortality in very old ICU patients (≥ 80 years) was around 22% and 30-day mortality was approximately 30–33% [1, 6].

Temporal trends suggest improving outcomes overall. Large registry data demonstrate declining mortality in very old ICU patients over time, particularly in unplanned admissions, suggesting advances in critical care delivery and patient selection [4].

### Illness severity

The degree of critical illness is independently associated with mortality, with an HR of 1.15 and an OR of 1.07 for each incremental increase in SOFA score [1, 7]. Unplanned (emergency medical or surgical) ICU admissions have a 30-day survival rate of ~ 60% compared with > 90% in elective ICU admissions [6]. Whilst selection for elective surgery explains this bias, the difference is important to note when making early assessments in the ED.

Use of organ support therapies is common but varies by frailty and clinical trajectory. Across cohorts, approximately half of patients receive vasoactive drugs, around 50% invasive ventilation, and 8–10% renal replacement therapy [6]. Frail patients are less likely to receive invasive ventilation and more likely to have treatment limitations imposed early in their ICU stay, reflecting both clinical judgement and patient-centred decision-making [7].

### Longer-term outcomes

However, longer-term and patient-centred outcomes are in many ways more important than mortality within this patient group. Survivors frequently experience significant functional decline and reduced health-related quality of life (HRQoL). Living with frailty strongly predicts poorer HRQoL following recovery from critical illness [9, 10]. Studies show persistent impairments in physical, social, and general health domains at 6 months following ICU admission, even when gradual recovery occurs [6, 11]. Premorbid functional status is strongly associated with survival, with poorer baseline HRQoL predicting mortality [12].

## Initial assessment of older people with critical illness: what is different?

Critical illness in older adults occurs within a context of frailty, multimorbidity, polypharmacy, and altered physiology, all of which influence clinical presentation, recognition, management, and outcomes. This section outlines how critical illness differs in older people and highlights practical adaptations required to deliver effective, realistic, and person-centred critical care for older people.

### ABCDE and F?

The ABCDE approach is well embedded in emergency care and offers a structured approach to the emergency assessment of patients of any age. It is standardised, well understood, and has strong external validity. But does it work for older people? We examine here how the ABCDE approach might vary in the context of frailty, and how emergency responders may need to adapt their approach.

### Airway and breathing

With increasing age, a decrease in elastic tissues reduces pharyngeal support, predisposing older people to upper airway obstruction. Dentures are common but should be left in situ to enable a better fit for ventilation masks. Intubation might be more challenging due to confusion, whether delirium or dementia. Ventilation pressures might need to be higher due to a reduction in the intrinsic elastic recoil of the lung parenchyma. Specifically in the context of stealth trauma, multiple rib fractures are more common, potentially compromising chest wall movements. Comorbidities such as chronic obstructive pulmonary disease mean that hypercarbia is more likely. Sedative agents such as propofol will need dose reductions given the altered pharmacodynamics and pharmacokinetics.

### Circulation

Age-associated alterations in cardiovascular physiology should be anticipated, including decreased beta-adrenergic responsiveness, conduction abnormalities, arrhythmias, and labile blood pressure—a form of autonomic failure. As with lung anatomy, increased ventricular stiffness is common, meaning that cardiac output is especially sensitive to ventricular pre-load/venous filling. But many older people will be volume deplete in the context of critical illness, related to poor oral intake in the preceding days, as well as the effect of commonly prescribed medications, such as diuretics. Clinicians tend to be nervous about overprescribing fluid to older people for fear of pulmonary oedema, yet the opposite is true. Guideline-recommended early fluid resuscitation (e.g. 30 ml/kg) has not been associated with excess pulmonary oedema in older cohorts [8, 9]. Close reassessment and early escalation for vasoactive support are essential when the response to fluid resuscitation is inadequate.

### Disability

Pre-existing disability, as well as (sometimes sub-clinical) cognitive impairment and cerebrovascular disease, is common, making distinguishing pre-existing neurological deficits from new deficits more challenging. The Glasgow Coma Score in people with dementia has dubious validity. Presbyopia, senile miosis, presbycusis, premorbid neuropathy, and reduced movement due to degenerative diseases further confound neurological assessment.

### Exposure

Homeostatic failure is to be expected in older people living at more advanced levels of frailty, leading to issues such as hypothermia in the face of even seemingly innocuous insults being common. Occult injuries can be easily missed if the assessor is not attuned to the phenomenon of stealth trauma. With the increasing use of anticoagulants and antiplatelet medication, combined with capillary fragility in older people, bleeding can be common and easily missed, for example, rectal sheath haematomas.

### F: Frailty-attuned geriatric critical assessment using the 5Ms

Having given examples of how the ABCDE approach might need to be adapted to meet the needs of older people living with frailty, it appears that the addition of F for frailty might be a useful consideration: ABCDE–F, the presence of frailty prompting assessing clinicians to adapt their approach [13].

Frailty is defined as an age-related physiological state with increased vulnerability to stressor events as a result of decreased physiological reserves and deregulation of multiple biological systems [14]. In research studies it is usually measured using the accumulation of deficit approach—a frailty index (FI) [15] or a phenotypic approach (Fried) [16]. In clinical practice, frailty should be explicitly identified using validated instruments such as the Clinical Frailty Scale (CFS) [17]. The CFS differs from the FI or phenotypic approaches, but ‘taps into’ the same domains, and has the added advantage of being quick, simple, and easy to use [18] and a strong predictor of mortality, length of stay, and readmissions in emergency settings [19], including trauma [20, 21]. Assessment must reflect baseline function 2 weeks prior to presentation, rather than the current state.

If the reader is now persuaded that measuring frailty might play a useful role in the assessment of critically ill older people, he or she will want to know more on how to operationalise this awareness. We offer the 5Ms model as a useful initial structure for a more holistic assessment.

The 5Ms framework (Mind, Mobility, Medications, Multicomplexity, and Matters Most) provides an accessible reference model for holistic and yet rapid geriatric assessment in acute situations. Its value lies in integrating comprehensive clinical consideration with reorientation of the encounter around the person as a whole rather than their diagnosis in isolation.

#### Mind

Cognitive and psychiatric domains are among the most frequently overlooked dimensions of acute assessment in older people: delirium is unrecognised in as many as 50% of acutely admitted older people [22]. Delirium is a medical emergency with potentially serious long-term sequelae, including accelerated cognitive decline and post-traumatic stress [23]. Its hypoactive form is particularly commonly missed, perhaps being mistaken for sedation or depression, which may explain its association with increased mortality. Systematic delirium screening during triage, for example using the 4AT instrument (www.the4at.com), adds minimal time to the assessment process while providing high sensitivity for detection [24, 25].

Dementia, depression, and anxiety are prevalent comorbidities in this population and each can significantly alter symptom presentation, communication, decision-making capacity, and recovery trajectory. A frailty-attuned assessment must therefore incorporate a baseline cognitive assessment (at minimum, using a screening tool). The comprehensive clinician will consider how the person’s mind might be reflecting, influencing, or masking the apparent physical presentation.

#### Mobility

Sarcopenia and deconditioning increase vulnerability to adverse outcomes during and after critical illness. These often manifest and can be identified during early assessment as impaired gait or balance, themselves all too frequently contributing to emergency care needs through falls. Where feasible, such assessments can inform understanding of the functional trajectory and identify impairments that will shape rehabilitation needs and safe discharge planning. In many situations it is practical and appropriate to represent mobility in the initial problems list and management plan, considering interventions or referrals aimed towards maintenance of current independence, limitation of deconditioning, and prevention of future falls. A simple test of mobility is the Timed Up and Go Test, which not only assesses mobility, but also screens for occult limb injury in those people able to complete it [26].

#### Medications

Polypharmacy is near-ubiquitous among older people living with frailty or multimorbidity, and medication-related harm is a major contributor to acute presentations in this group [27]. A frailty-informed assessment reviews the current medication list critically, seeking both direct and indirect contributions to the acute episode. Considerations include anticholinergic load, drug interactions, nephrotoxic effects, sedation (as an intention or consequence), or hypotensive effects. Antithrombotic and anticoagulant agents require specific attention in the context of acute haemorrhage or trauma, and their effects may require reversal in emergencies. Consider also the potential contributions by withdrawal of substances, prescribed or otherwise, to the current situation.

The acute admission represents an opportunity for structured medications review and deprescribing consideration, recognising that symptom burden and treatment goals may have shifted over time since drugs were initiated. Implicit in this domain is awareness that prescribing during acute illness must account for altered pharmacokinetics and pharmacodynamics associated with ageing and frailty.

#### Multicomplexity

People living with frailty characteristically experience multiple concurrent and interacting conditions, each with its own treatment regimen, specialist involvement, and monitoring requirements. Multimorbidity (the presence of two or more chronic health conditions) is virtually ubiquitous. This multicomplexity challenges single-organ, single-diagnosis approaches to emergency management and demands that the practitioner assume a more comprehensive stance. In around one in ten older people, the acute presentation itself is non-specific (for example, fatigue, confusion, or a general deterioration in function) resisting conventional diagnostic pathways and yet carrying a high risk of adverse outcomes [28, 29].

A frailty-attuned assessment seeks to understand multicomplexity in the patient’s whole situation: not only which medical conditions are active, but how the acute episode fits within a broader picture and trajectory. This requires access to a longitudinal clinical record where possible, and ideally early integration of multidisciplinary professional care. Comprehensive geriatric assessment (CGA) operationalises this domain at the highest level, and with adequate resources can be implemented in the emergency setting [30].

#### Matters most

The fifth and arguably most pivotal ‘M’ is the explicit elicitation of what the person themselves finds important: their values, goals, preferences, and concerns. In contrast to conventional history-taking oriented around diagnosis and treatment, Matters Most directs attention to the lived experience of illness and the outcome goals that are meaningful to the individual. International consensus work found that merely *extending* life may not be the highest priority for older people living with frailty [31]. Indeed, we appreciate that even younger and fitter people might place greater value in remaining independent and preserving quality of life, prioritising these above longevity. Meanwhile enablement, encompassing the desire to gain health knowledge and to feel safe in one’s situation, emerges as a consideration of particular importance [32, 33]. These preferences are frequently poorly documented and rarely guide acute management decisions, despite their capacity to ground complex decisions and reduce both professional uncertainty and patient distress [34, 35].

Understanding Matters Most is not simply a component of initial assessment; it functions as the moral and clinical anchor for subsequent decision-making. In the context of critical illness, this dimension becomes inseparable from questions of treatment escalation, intensive care admission, and the appropriate balance between curative and comfort-oriented care. These themes are explored in the following section. Effective elicitation of patient goals requires not only a clinician willing to ask, but a system designed to facilitate, communicate, and enact those conversations across care transitions.

## Person-centred geriatric critical care

Assessment to inform consideration of life-sustaining critical care takes place in a wider context of mounting recognition that survival alone is an insufficient measure of meaningful outcome. A 2022 Lancet Commission report drew attention to the medicalisation of dying, highlighting how healthcare systems are structurally oriented towards the control and deferral of death rather than the facilitation of good death, resulting in widespread non-beneficial treatment in the final months and weeks of life [36]. Around one-third of people receive potentially non-beneficial interventions as they approach the end of life, and among those who die, half receive such treatments within the final two days of life [37]. These patterns call for a fundamental reorientation of frailty-attuned decision-making towards person-centred and value-based frameworks.

Studies of person-centred outcomes following critical care are few and far between, with the vast majority of the literature focusing upon service measures, destination outcomes, and mortality. This is most likely because such outcomes are relatively easy to ascertain—but how relevant are they to older people with critical care needs?

### The case for value-based and goal-oriented care

A goal-oriented care paradigm was articulated over a decade ago as an alternative to the prevailing framework of care outcomes judged by mortality, readmission, and length of stay [38]. Instead, individual health goals encompassing functional independence, symptom control, social participation, and quality of life become the primary reference for care planning and evaluation. Goal-oriented approaches simplify shared decision-making by providing a principled basis for comparing treatment options against what the person values, rather than defaulting to treatment escalation as a proxy for good care [39]. Qualitative research has illuminated the nuance of these preferences: older people living with frailty increasingly find meaning in 'smaller things' in life, such as close relationships, familiar environments, maintained cognitive presence [33].

Value-based care extends this framework to consider the proportionality of treatments relative to their likely benefit. This does not imply a nihilistic approach to the critically unwell older person; rather, it insists that the probability of meaningful recovery, in terms the patient would recognise as meaningful, is weighed against the burden of intervention.

Recent consensus-based recommendations from the European Society of Intensive Care Medicine (ESICM) for the management of 'very old' patients (aged 85 and above) in intensive care explicitly endorse this person-centred approach to decision-making around critical care admission [40]. Recommendations include avoiding the use of chronological age alone as an admission criterion; systematically eliciting patients’ preferences regarding acceptable functional outcomes and quality of life; incorporating advance directives where available; recognising frailty, multimorbidity, and functional impairment as key prognostic modifiers; tailoring treatment intensity to both acute illness and premorbid status; avoiding age-based treatment limitation; weighing the time-dependent burden of interventions against likely benefit; and promoting continuity of care across hospital and community settings.

Person-centred critical assessment is not a checklist but an ethical orientation, positioning the older person’s humanity, voice, and goals at the centre of the clinical encounter. Collectively, these principles position frailty-attuned, preference-sensitive assessment as the foundation for ICU decision-making in very old adults.

### Pragmatic decision-making in critical situations

In critical situations, goals of care conversations require practical skills in shared decision-making, which encompasses not only communication but also the capacity to synthesise prognostic information, present options that align with patient values, and make recommendations that reflect clinical experience alongside individual goals [41].

When decision-making capacity is impaired, as it frequently is in critical presentations, a person-centred approach adapts rather than collapses. Available evidence about likely outcomes is integrated with surmised knowledge of the individual’s values, beliefs, and preferences by drawing on advance care plans, those close to the person, and the documented record of previous encounters [42]. This approach is grounded in the ethical principles of autonomy and beneficence: respecting the person’s right to have their values honoured and acting in their genuine interest rather than defaulting to maximal treatment as an expression of clinical virtue.

Uncertainty is a professional constant in critical care. Prognostic uncertainty is especially pronounced in older people with frailty, in whom the trajectory of recovery is harder to predict and in whom standard physiological severity scores may underestimate or misrepresent risk [43]. High-complexity and high-stakes decisions may exceed the scope of standard protocols and guidelines. Importantly, tolerance (or management) of clinical uncertainty does not increase linearly with professional experience, suggesting that decision-making dispositions reflect individual as much as institutional factors, and that supporting clinicians in navigating uncertainty deserves attention in professional development [44].

Shared decision-making is a trainable and deployable skill even in time-pressured environments, and its adoption need not require prolonged consultations. Understanding what matters most, as elicited through the 5Ms framework, provides the essential foundation for pragmatic decisions that are both clinically sound and consistent with values. Advance care planning, when effectively communicated and available at the point of need, can support and legitimise these decisions. However, the absence of a documented plan does not foreclose a person-centred approach; rather, it places a greater responsibility on the clinician to enquire, listen, and proceed with due regard for what can be reasonably inferred about the patient’s values. Recognising futility and avoiding non-beneficial treatments represent not the withdrawal of care but its highest expression: directing attention and resources towards comfort, dignity, and the outcomes that the person themselves would have chosen.

## Conclusion

Critical illness in older people requires a frailty-informed, holistic, and person-centred approach. Recognition of atypical presentations, contextual interpretation of physiology, early goal alignment, and decisive treatment when appropriate are essential to optimise outcomes while avoiding both ageism and therapeutic nihilism. Moreover, the evidence base to date relates to improvements in outcomes that may have limited relevance to older people living with frailty and who have critical care needs. In critiquing the structure, process and outcomes pertaining to critical care, we propose here a paradigm shift to person-centred, outcome-driven, frailty-attuned critical care.

## Data Availability

No data are available
